# Implications of iodine deficiency by gestational trimester: a systematic review

**DOI:** 10.20945/2359-3997000000289

**Published:** 2020-08-24

**Authors:** Aline Carare Candido, Francilene Maria Azevedo, Almeida Abudo Leite Machamba, Carina Aparecida Pinto, Sílvia Oliveira Lopes, Mariana de Souza Macedo, Sarah Aparecida Vieira Ribeiro, Silvia Eloiza Priore, Sylvia do Carmo Castro Franceschini

**Affiliations:** 1 Universidade Federal de Viçosa Departamento de Nutrição e Saúde Viçosa MG Brasil Departamento de Nutrição e Saúde, Universidade Federal de Viçosa (UFV), Viçosa, MG, Brasil

**Keywords:** Implications, iodine deficiency, pregnancy

## Abstract

As pregnant women are susceptible to changes in iodine, which can cause miscarriage, goiter, thyroid nodules, hypothyroidism, in addition to fetal neurological impairment or development. The aim of this study was to verify the implications of the iodine alteration in each gestational trimester and its consequences of physiological justification. The review was based on PRISMA. Searching for articles that took place in March 2020 without delimiting data. As bases consulted were the Clinical Trials, Cochrane Library, Lilacs and Medline (PubMed). The descriptors were combined as follows: "pregnancy" AND "iodine deficiency". Articles that addressed iodine deficiency and its implications were included. The selection followed the steps of reading the titles, abstracts and full articles. To assess the methodological quality of the studies, the STROBE Instruction instrument was used. The research resulted in 1,266 studies and 11 were included. In assessing methodological quality, the lowest score was and the maximum 20. According to studies, the fourth most affected by iodine loss are the second and third, it is possible to increase the volume and pneumatic nodules, subclinical hypothyroidism, pre-eclampsia, among others. The damages caused by iodine deficiency in the first or second trimester are still reversible, therefore, they need to be diagnosed early, to guarantee an iodic homeostasis and prevent damage to the health of the mother-child binomial.

## INTRODUCTION

Gestation is a period of two great vulnerabilities: biological, because there is intense growth and fetal development and; nutritional, due to increased energy requirements (1). Therefore, it is necessary to make pregnant women aware of the importance of adequate feeding, in order to prevent health problems caused by nutritional deficiencies (2).

Iodine is an essential micronutrient for the regulation of metabolic processes and for the synthesis of thyroid hormones responsible for the development of the central nervous system in the embryonic period (1). Pregnant women are more susceptible to the deficiency of this mineral due to the transfer of hormones to the fetus and increased glomerular filtration, leading to iodine loss in the urine (2).

Iodine deficiency during pregnancy can lead to spontaneous abortion, goiter, thyroid nodules, hypothyroidism, and compromise fetal neurological development. Thus, there is an increase with expenses on health and education, generating social and economic losses for the countries (3).

Studies have already evaluated the damage caused by insufficient iodine intake, but there is not yet a compiled study in the literature to highlight the implications that iodine deficiency can cause during pregnancy. This systematic review was based on PRISMA and sought to answer the guiding question “what are the implications of iodine deficiency in each gestational trimester?” Therefore, the objective of this review was to verify the implications of iodine deficiency in each gestational trimester and their respective physiological justifications.

## METHODS

It is a systematic review, based on the recommendations of the Preferred Reporting Items for Systematic Reviews (PRISMA) (4). The guiding question was “What are the implications of iodine deficiency in each gestational trimester?” To define this question, the PECOS criteria were used, as shown in Table 1.

**Table 1:** PECOS criteria used to define the guiding question of the systematic review

Criterion	
P	Pregnant women every trimester
E	Iodine deficiency in the first, second and third gestational trimester
C	Without comparison
O	Consequences of disability in each trimester = causes and physiology
S	Observational and clinical

We have registered the article at the International Prospective Register of Ongoing Systematic Reviews (PROSPERO) with identification: CRD42019129885.

The search occurred in March 2020 without date delimitation. The databases consulted were Clinical Trials, Cochrane Library Center, Latin American and Caribbean Literature in Health Sciences (Lilacs) and Publisher Medline (PubMed). The descriptors determined by the Health Science Descriptor system (DeCS) were combined as follows: “pregnancy AND “iodine deficiency” in English, Portuguese and Spanish, using the terms “human” and “women” as filters.

We have included articles that addressed iodine deficiency and implications during pregnancy. We have excluded systematic review papers, experimental papers, government documents, studies on other micronutrients that did not include iodine, iodine supplementation/fortification, salt iodization monitoring, thyroid function assessment (without considering the deficiency of iodine), other age groups and other subjects.

The selection of articles was conducted by two researchers, independently. In case of disagreement regarding the inclusion or not of a particular study, a third researcher was consulted. The titles, abstracts and full articles were read; if they did not meet the inclusion criteria, the studies were eliminated.

After the selection, we conducted a qualitative synthesis of the articles included in this review, systematizing the most relevant information, such as: authors, study design, main results and conclusions.

In order to assess the methodological quality of the articles, we used the Strengthening the Reporting of Observational Studies in Epidemiology (STROBE Statement) instrument (5), which contains a list of 22 items to verify the information that should be present in the title, abstract, introduction, methodology, results and discussion of scientific articles. Responses were scored as “1” (when the criterion that characterized quality was present) or “0” (when the criterion was absent). The studies were classified in scores from zero (worst quality) to 22 (best quality).

## RESULTS

**Figure 1:**
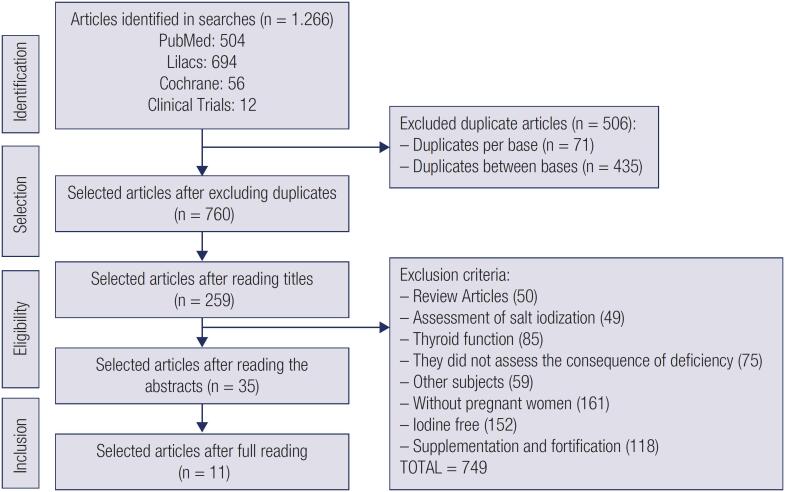
Flowchart of the process of identification and selection of the articles included.

Source: PRISMA (4).

The search resulted in 1,266 studies. After excluding the duplications by base and between the bases, 760 remained. Afterwards, we made the selection, through the steps of reading the titles, abstracts and full article. At the end, we included 11 articles (Figure 1).

Of the included studies, 63.6% were of longitudinal design, 27.3% were cross-sectional and 9.1% were control cases. The years ranged from 2009 (8,9) to 2018 (3) and the sample size from 57 (8) to 5256 (9). The main results of the studies are described in Table 2.

**Table 2:** Main results of selected studies for the systematic review

Authors/Year	Local	Design	Sample size (n)	Gestational trimester analyzed	Main results for pregnant women
Alvarez-Pedrerol and cols., 2009 (6)	Spain	Cohort	239 pregnant and puerperal women	1^st^ e 3^rd^ trimester GA: -	Pregnant women with iodine deficiency were more likely to have children with low birth weight and SGA. However, the ones with excess of iodine of having children with significant increase of weight.
Harun-Or-Rashid and cols., 2009 (7)	Bangladesh	Cross-sectional	355 adolescents, 263 pregnant women and 395 nursing	2^nd^ e 3^rd^ trimester GA: ≤12 weeks (APG: 6.3 months)	Of the pregnant women, 44.4% were anemic; 39.4% deficient in iodine, so they were more likely to have diarrhea/dysentery, pneumonia and ear infection.
Bath and cols., 2013 (10)	England	Longitudinal	958 pregnant women with their respective children (8 and 9 years).	1^st^ trimester GA median: 13 weeks (IQR: 9-12)	Pregnant women with light and moderate iodine deficiency were more likely to have children with lower verbal IQ, accuracy and reading comprehension.
Ghassabian and cols., 2014 (1)	Netherlands	Cohort	1,525 pregnant women and their respective children (6 years)	2^nd^ trimester GA mean: 13.3 (CI%: 6.0-17.9)	Association between low UIC in gestation and sub-optimal non-verbal IQ of children. However, after adjustment, low maternal UIC was not associated with nonverbal IQ in children.
Joshi and cols., 2014 (9)	India	Cross-sectional	5,256 pregnant women	1^st^ e 2^nd^ trimester GA: <15 weeks	Pregnant women with iodine deficiency also had iron deficiency (16.4%). As TSH, levels increased from 1^st^ to 2^nd^ trimester, FT4 decreases.
Vidal and cols., 2014 (11)	Mexico	Cross-sectional	212 pregnant women	1^st^, 2^nd^ and 3^rd^ trimester GA: -	In pregnant women with low iodine deficiency in all trimesters, oxidative stress was higher, with reduction of total antioxidant status and SOD activity.
Sahin and cols., 2014 (12)	Turkey	Longitudinal	83 pregnant and puerperal women	1^st^ and 3^rd^ trimester GA: -	Multiparous pregnant women presented thyroid nodules (50%). Nodule volume increased during pregnancy, with the largest diameter detected in the 3^rd^ trimester.
Charoenratana and cols., 2016 (13)	Thailand	Longitudinal	384 pregnant women in the 1^st^ trimester, 325 in the 2^nd^ and 221 in the 3^rd^	1^st^, 2^nd^ and 3^rd^ trimester GA: -	Pregnant women with iodine deficiency had a higher risk for restriction of fetal growth, prematurity and low birth weight when compared to pregnant women with adequate iodine nutritional status.
Cuellar-Rufino and cols., 2017 (8)	Mexico	Case control	57 pregnant women 20 cases e 37 control	3^rd^ trimester GA: -	Pregnant women with iodine deficiency had hypertension (70%). Iodine deficiency during pregnancy was associated with lower SOD activity, lower total antioxidant status and higher oxidative stress.
Xiao and cols., 2018 (2)	China	Cohort	1,569 pregnant women	1^st^ trimester GA: 4-12 weeks	Pregnant women with light iodine deficiency were more likely to have gestational diabetes mellitus and had a higher prevalence of diabetes and placental abruption. Pregnant women with excess iodine were more likely to have a baby with higher birth weight.
Torlinska and cols., 2018 (3)	England	Longitudinal	3,140 pregnant women and 42 women with abortion or child loss up to 1 year	1°, 2° e 3° trimester GA: -	Pregnant women with iodine deficiency and adequate nutritional status did not present differences in the incidence of pre-eclampsia, hypertension, gestational diabetes, glycosuria, anemia, postpartum hemorrhage, preterm delivery and SGA babies.

GA: gestational age; APG: average period of gestation; IQR: interquartile range; CI%: confidence interval; SGA: small for gestational age; IQ: intelligence quotient; UIC: urinary iodine concentration; FT4: free thyroxine; SOD: superoxide dismutase.

**Figure 2:**
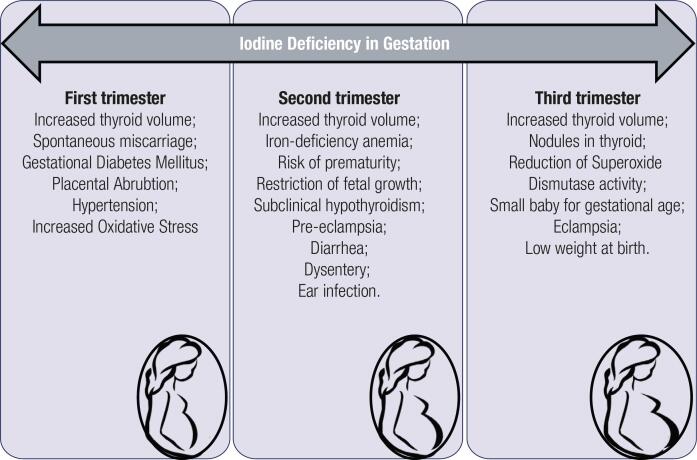
Summary of the implications caused by iodine deficiency in each gestational trimester observed in the articles included in the systematic review.

The consequences of iodine deficiency differed according to the gestational trimester. Figure 2 presents, according to the included studies, the main damages identified when the pregnant woman has insufficient intake of iodine.

In the assessment of the methodological quality of the studies according to STROBE Statement, the lowest score was 15 (27.3%) and the highest 20 points (9%), indicating good methodological quality among the included studies and a satisfactory level of reliability for the presented results.

## DISCUSSION

Iodine deficiency disorders (IDD) occur when the pregnant woman has insufficient iodine intake, which results in a lower production of thyroid hormones, negatively affecting the child's muscle, heart, liver, kidneys and brain development (3).

**Figure 3:**
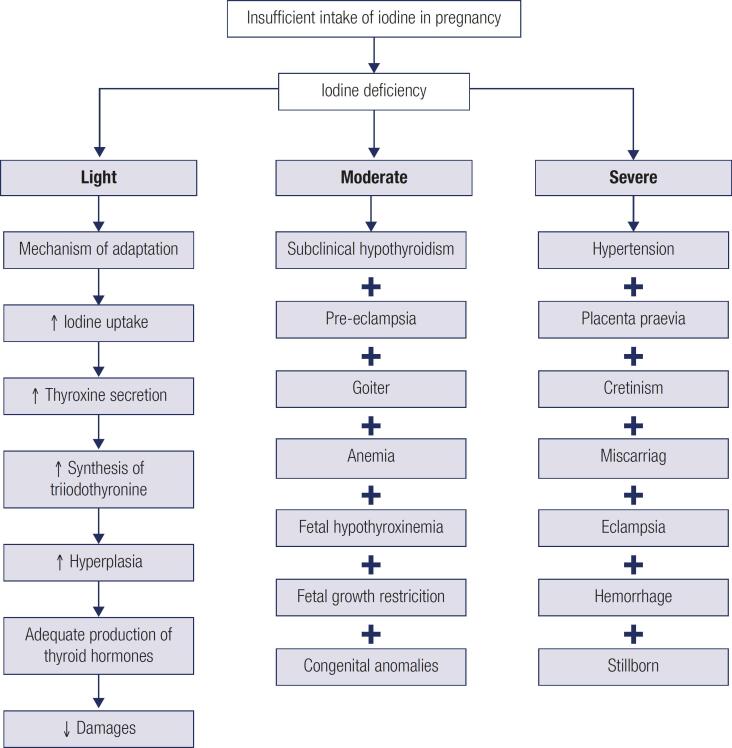
Implications for the pregnant woman according to the degree of iodine deficiency (2,11,13,14).

The implications of iodine deficiency in pregnancy differ according to the degree of deficiency and gestational trimester. In light deficiency the process is continuous, while the others present a summation effect and independent unfolding (Figure 3). This division is essential to guide strategies to control and prevent these health implications.

### First trimester

In the first trimester of gestation, the blood flow and glomerular filtration increase resulting in loss of iodine in urine. The organism, as an adaptation mechanism, increases the secretion of the hormone human chorionic gonadotrophin and thyroid stimulating hormone (TSH), which induces the thyroid to increase the concentration of free thyroxine (T4), guaranteeing the production of thyroid hormones (15).

The light iodine deficiency acted as a risk factor for diabetes mellitus and placental abruption in a study conducted in China. According to the authors, the lack of iodine in the organism induces the increase of TSH secretion in plasma, producing an antagonistic effect on insulin, generating hyperglycemia; which is associated with increased oxidative stress, which is a risk factor for placental abruption (2).

These results corroborate a study conducted in Mexico, where oxidative stress was higher in pregnant women with iodine deficiency in the first trimester, with reduction of antioxidant activity and superoxide dismutase (SOD). Due to oxidative stress, there is an increase in the production of free radicals, which cause hypertension and endothelial dysfunction, which can lead to miscarriage and pre-eclampsia (11).

Severe iodine deficiency, in addition to affecting the mother, also causes harm to her child. A longitudinal study conducted in England with pregnant women found the influence of iodine deficiency on the cognitive ability of their respective children. Children of descendants of women with severe disabilities presented results below the intelligence quotient (IQ) ideal, presenting speech difficulties at 8 and 9 years of age in reading accuracy and comprehension. The low socioeconomic level of the participants associated with insufficient iodine intake may have been determinant for these detected results (10).

Some authors suggest that iodine supplementation should ideally be performed in the first trimester, since it would be possible to maintain iodine reserves in the body, guaranteeing the production of thyroid hormones and avoiding damage to maternal and child health (2,10,11).

### Second trimester

In the second trimester of pregnancy, TSH levels return to normal and fetal development becomes indirectly dependent on the maternal thyroid because the fetal thyroid, even when immature, also begins to produce hormones (15).

In a study conducted in India, 16.4% of pregnant women with low iron intake showed an increase in corpuscular volume and glomerular filtration, being diagnosed with iron and iodine deficiency (9). In Bangladesh, 39.4% of anemic pregnant women had iodine deficiency and, due to the nutritional vulnerability caused by the association of these two deficiencies, pregnant women were more likely to have diarrhea/dysentery, pneumonia and ear infection (7).

In Thailand, a study showed that iodine deficiency was higher in the second trimester, increasing the risk of pre-eclampsia, fetal growth restriction, low birth weight and prematurity (13).

Ghassabian and cols. assessed the association between iodine deficiency during pregnancy and IQ in their 6-year-old children. The authors observed an association between insufficient iodine intake and sub-optimal nonverbal IQ in children. However, after adjusting for confounders, there was no association between non-verbal IQ and deficiency (1).

According to the studies, mild iodine deficiency, if diagnosed in the second trimester of pregnancy, it is still possible to resolve the implications caused by the deficiency through iodine supplementation (1,7,9,13).

### Third trimester

In the third trimester of gestation, thyroid hormone-dependent neurogenesis is still underway and if iodine deficiency is present since the first trimester, the implications may be irreversible (8).

A study conducted in Spain showed that pregnant women with iodine deficiency in the third trimester are more likely to have a low weight newborn and Small for Gestational Age (SGA), due to fetal growth restriction and low production of thyroid hormones (6).

Iodine has an important antioxidant function. Its deficiency can increase the levels of oxidative stress, causing the development of complications during pregnancy and hypertensive disorders. In a study conducted in Mexico, iodine deficiency during gestation was associated with hypertension, lower SOD activity and higher oxidative stress. According to the authors, these results are worrying because they can cause placental abruption, eclampsia and even hemorrhages (8).

The main target of iodine deficiency in pregnancy is the thyroid. Due to the mechanism of autoregulation, there is an increase in the secretion of TSH that overestimates this gland, leading to an increase in thyroid volume and the formation of nodules (12).

A study conducted in Turkey, 50% of iodine deficient multiparous women had thyroid nodules that were increasing in volume during pregnancy, with the largest diameter detected in the third trimester; however, the quantitative of nodules remained the same. According to the authors, these results may be justified by the fact that pregnant women with nodules are older, have more children and have a worse socioeconomic status than those without nodules. Also, because of the compression that the placenta exerts under the bladder, the pregnant women increase the urinary frequency in the last trimester, leading to a negative balance of circulatory iodine, causing thyroid nodules (12).

On the other hand, a study in England that analyzed the impact of iodine deficiency in all trimesters of pregnancy found no difference between the incidence of pre-eclampsia, hypertension, gestational diabetes, glycosuria, anemia, preterm birth, SGA babies and hemorrhage postpartum among pregnant women with deficiency and adequacy of iodine status (3).

### Strengths and limitations

Strengths: the work was conducted in different regions, representativeness of the samples and the review was a compilation of information subdivided according to the trimesters, which will contribute to clinical practice and generation of evidence, which will be important for diagnosis and consequent decision making, thus avoiding future harm to the child.

The limitation was that only 63.6% of the included studies were of a longitudinal design, that is, the other studies did not allow to verify the causality, only to identify associations between iodine deficiency and the implication identified in that period of pregnancy.

### Final remarks

The consequences caused by iodine deficiency vary according to the trimester of gestation. The most affected trimesters in the studies assessed were the second and third, probably due to the neurological development of the fetus that increase the nutritional need of the mother.

However, if such implications still occur in the first or second trimester, there is a possibility of reversal. Therefore, the early diagnosis of iodine deficiency during the gestational period is essential, since it provides guidance for adequate intake, either dietary or supplementation, guaranteeing iodine homeostasis and preventing damage to maternal and child health.
